# Assessing psychosocial risk factors in children with Sickle Cell Disease

**DOI:** 10.1186/s12913-025-12266-y

**Published:** 2025-01-18

**Authors:** Nicole Frey, Julia E. LaMotte, Jillian R. Bouck, Lauren Fancher, Genese T. Parker, Allie Carter, Seethal A. Jacob

**Affiliations:** 1 Indiana University School of Medicine, 410 W 10th St, Suite 2000A, Indianapolis, IN 46202 USA; 2 Division of Pediatric Hematology, Oncology and Stem Cell Transplant, Riley Hospital for Children, 410 W 10th St, Suite 2000A, Indianapolis, IN 46202 USA; 3 Indiana University-Purdue University Indianapolis, Indianapolis, IN 46202 USA; 4 Division of Children’s Health Services Research, 410 W 10th St, Suite 2000A, Indianapolis, IN 46202 USA; 5 Department of Biostatistics and Health Data Science, Indianapolis, IN 46202 USA

**Keywords:** Sickle cell disease, Psychosocial factors, Social determinants of health, Caregiver stress, Access to health care

## Abstract

**Background:**

Individuals with Sickle Cell Disease (SCD) are a minoritized and marginalized community that have disparate health outcomes as a result of systemic racism and disease-related stigma. The purpose of this study was to determine the psychosocial risk factors for families caring for children with SCD at a pediatric SCD center through use of the Psychosocial Assessment Tool (PAT), a validated caregiver-report screener.

**Methods:**

The PAT was administered annually during routine clinical visits and scored by the SCD Social Worker to provide tailored resources to families. The PAT stratifies scores into 3 categories of psychosocial concern: Universal, Targeted, Clinical. PATs administered between September 2021-December 2022 were analyzed.

**Results:**

Two hundred twenty-five PATs were included for analysis. Most caregivers identified as Black, single Women over 21 years old with a high school degree or more. The average patient age was 8.2 years (0–22 years). Sixty-seven percent of PATs fell into the Universal category. Dyads that scored in the Targeted or Clinical categories were more likely to report financial hardship, caregiver mental health concerns, and family stressors (*p* < 0.001). Nearly 50% of all families reported some form of financial difficulty, including almost 40% in the Universal category.

**Conclusions:**

Universal implementation of a psychosocial risk screener identified financial challenges for many families, as well as caregiver burden and mental health concerns, allowing for timely resource support. However, overall risk for many of these families was categorized as Universal or low risk, indicating that distribution of resources and support cannot be based on PAT category alone.

## Introduction

Despite being the most common inherited blood disorder in the world, Sickle Cell Disease (SCD) predominantly impacts minoritized communities in the United States, where systemic racism and structural inequities in education and medicine have notably contributed to disproportionate access and the neglect of this population within healthcare [1, 2]. In a study by Boulet et al., children with SCD experienced longer than average emergency department wait times, difficulty with transportation, and insufficient availability of primary care providers compared to children without SCD [3]. Additionally, comprehensive SCD centers are more commonly located in major metropolitan areas, creating additional access challenges for families living in rural or under-resourced areas [4, 5].

Social determinants of health (SDoH) encompass the external factors affecting health, including the environments in which people live as well as their basic unmet social needs such as financial stress, food insecurity and housing instability [6]. SDoH have a profound impact on health and wellbeing and are also largely responsible for health inequity [6, 7]. This can lead to poor treatment adherence, inadequate disease management, and increased acute care utilization in patients with SCD [7–10]. Additionally, these factors place heightened stress on caregivers managing their child’s chronic condition, impacting caregiver mental health, resiliency, and family functioning [11–13].

The Pediatric Psychosocial Preventative Health Model (PPPHM), initially described by Kazak in 2006, is a framework that integrates the range of social and psychological factors that impact a family’s ability to adapt to having a child with a chronic illness [14]. This psychosocial framework consists of a tri-level risk stratification facilitating a comprehensive screening strategy within healthcare settings to identify social and psychological risk elements that could influence a child's well-being. Given the health disparities seen in SCD and its known impact on caregiver stress and mental health, the PPPHM serves as a critical framework for understanding opportunities for intervention that may ultimately mitigate the impact of psychosocial risk factors on child and family health.

A broad assessment of psychosocial risk factors for families of youth with SCD that evaluates SDoH, as well as child behaviors and attention concerns, caregiver mental health, and traumatic stress response, for example, allows for a more directed intervention that can ultimately improve disease management. The Psychosocial Assessment Tool (PAT) is a validated caregiver-report survey used to broadly screen for family psychosocial risk, which then categorizes risk based on the tiered PPPHM model [15]. Respondents map to one of three categories—Universal, Targeted, and Clinical—representing the degree of psychosocial intervention needed to support the patient/family. The PAT has previously demonstrated validity and utility in several studies with children and adolescents with chronic diseases, both in the ability to determine the level of psychosocial risk factors and ascertain which families need resources, but also in the ability to correlate with different measures such as healthcare utilization and quality of life [16–21]. To ensure validity in SCD specifically, the initial version of the PAT was modified based on feedback from caregivers of children with SCD by Reader, et al. [16, 17]. However, there have been few small cohort studies in SCD since that time, and none evaluating the impact of universal clinical application of the PAT [20–22].

The purpose of this study was to determine the degree of psychosocial risk factors for families cared for at a comprehensive pediatric SCD clinic in the Midwest with a large, geographically diverse catchment area through universal psychosocial screening.

## Methods

### Participants/process

Caregivers of children with SCD seen in comprehensive SCD clinic were asked to complete the PAT at their first clinic visit and then annually to identify changes in family needs and/or stressors. Patients were identified by the SCD Nurse Navigator and/or Social Worker prior to the clinic visit, and PATs were distributed at the time of check-in to the appointment. Families were instructed to complete the form by the Medical Assistant rooming the patient. All surveys were distributed in English, with an interpreter present for families whose Language is other than English. Results were reviewed by the team Social Worker, who followed up with families in-person or by phone based on needs identified and/or total PAT score. An Electronic Medical Record (EMR) template was created for the SCD Social Worker to document subscale scores, as well as total score/PAT Category and any resources provided, and the completed PAT was uploaded to the EMR. Any concerns about Child/Sibling Behaviors and/or Caregiver Stress were communicated to the SCD Psychologist.

### PAT measure

The PAT is a brief caregiver reported screener of family psychosocial risk that generates a total score based on seven domains or subscales (Family Structure/Resources, Social Support, Child Problems, Sibling Problems, Family Problems, Caregiver Stress Reactions, and Family Beliefs). The Family Structure/Resources subscale asks questions regarding caregiver education, transportation, as well as financial concerns. The Social Support subscale asks about various circles of support for the child, caregiver, and family. The Child and Sibling Problems subscales divide questions based on age (i.e. Under 2, Over 2), and includes questions about child/sibling behavior, mood, and trauma exposure. Families were able to answer questions in multiple age categories for siblings if relevant (e.g. Sibling Under 2 and Sibling Over 2). The Family Problems subscale assesses caregiver mental health, substance use, as well as trauma exposure. The Caregiver Stress subscale focuses on stress response. The Family Beliefs subscale asks about factors related to trust in the medical team and worldview.

PAT items are scored using prespecified criteria to indicate risk or no risk [15]. Items are scored as 1 (risk endorsed) or 0 (risk not endorsed). PAT subscale scores are determined by calculating the average of the binary items. The PAT total score is calculated by adding the subscale scores. Subscale scores can range from 0.00 to 1.00, while the total score can range from 0.00 to 7.00. The total score maps onto the PPPHM (Kazak, 2006) with three levels of risk—Universal (< 1.00), Targeted (1.00 and 2.00), and Clinical (> 2.00). Patients in the Universal category may have minimal social and psychological distress with or without protective factors that may buffer impact, whereas patients in the Clinical category have more severe levels of psychosocial distress and/or limited protective factors.

### Analysis

Retrospective PAT data from September 2021 to December 2022 were collected from the EMR. Descriptive statistics and comparisons were performed for the first instance of the PAT screener per family. Continuous data were summarized as means and SDs, and categorical data were summarized as frequencies and percentages. Subscale scores and outcomes of interest, along with demographic characteristics were compared between the Universal and Targeted/Clinical PAT categories. Categorical responses and demographics were compared by Chi-Square test. In cases of small sample size, a Fisher’s Exact test was utilized. Ordinal responses were compared by Mantel–Haenszel test. For numeric variables, one way ANOVA was used for Normally distributed variables, while Wilcoxon was used for non-Normally distributed variables.

For the subset who completed two PAT screeners during the study period, screeners were analyzed for differences between the first and second instances of the PAT. For Normally distributed differences, paired t-tests were performed. For non-Normal differences, signed rank tests were performed. For the ordinal categorical variable, Cochran-Mantel–Haenszel was used.

All analytic assumptions were verified. Data summaries were produced using R Statistical Software (version 4.1.0) and all analyses were performed using SAS version 9.4 (SAS Institute, Inc., Cary, NC, USA). A *p*-value of < 0.05 was used to define statistical significance. This study was considered exempt by the Indiana University Institutional Review Board.

## Results

A total of 265 PATs were administered during the study period. Fifteen were incomplete and not included in the analysis, and 4 families chose not to complete the PAT due to time limitations, resulting in 246 PATs completed by caregivers of 225 unique patients (i.e. 21 caregivers completed more than 1 PAT in this period).

### Demographics for unique patients

The unique patient sample consisted of 115 males and 110 females. The majority of patients (96%) were Black or African American. The average patient age was 8.2 years old with a range of 0–22 years old (Table 1). Approximately 83% of children were publicly insured. Fifty-four percent of caregivers reported their child was aware of their diagnosis, and 45% were not aware because they were too young. Only three patients did not know about their diagnosis because their family decided not to tell them. There were no significant differences between the Universal and Targeted/Clinical groups in child age, sex, ethnicity, race, or awareness of diagnosis.

**Table 1:** Demographics for unique patients and caregivers

	**Universal (*N* = 150)**	**Targeted/Clinical (*N* = 75)**	**Total (*N* = 225)**	***p* value**^**a, b, c, d**^
**Age at Survey**				0.309^d^
Mean (SD)	7.9 (6.0)	8.8 (5.9)	8.2 (6.0)	
Median (Q1, Q3)	6.7 (2.3, 13.5)	9.5 (2.8, 13.5)	7.3 (2.6, 13.6)	
Range	0.0—22.1	0.0—19.4	0.0—22.1	
**Child Sex**				0.509^a^
Male/Boy	79 (52.7%)	36 (48.0%)	115 (51.1%)	
Female/Girl	71 (47.3%)	39 (52.0%)	110 (48.9%)	
**Child Ethnicity**				0.720^b^
N-Miss	77	34	111	
Hispanic/Latino	5 (6.8%)	4 (9.8%)	9 (7.9%)	
Not Hispanic/Latino	68 (93.2%)	37 (90.2%)	105 (92.1%)	
**Child Race**				0.934^b^
N-Miss	2	0	2	
Asian	1 (0.7%)	0 (0.0%)	1 (0.4%)	
Black/African American	141 (95.3%)	73 (97.3%)	214 (96.0%)	
Multi-Racial	3 (2.0%)	2 (2.7%)	5 (2.2%)	
White/Caucasian	1 (0.7%)	0 (0.0%)	1 (0.4%)	
Other	2 (1.4%)	0 (0.0%)	2 (0.9%)	
**How long has your child been diagnosed with their current illness/injury?**				0.172^c^
N-Miss	14	6	20	
Less than 1 month	10 (7.4%)	2 (2.9%)	12 (5.9%)	
Less than 3 months	8 (5.9%)	2 (2.9%)	10 (4.9%)	
Less than 1 Year	9 (6.6%)	6 (8.7%)	15 (7.3%)	
1 year to 3 years	21 (15.4%)	11 (15.9%)	32 (15.6%)	
3 to 5 years	21 (15.4%)	8 (11.6%)	29 (14.1%)	
More than 5 years	67 (49.3%)	40 (58.0%)	107 (52.2%)	
**Does your child know about/understand his or her illness and its treatment?**				0.110^b, e^
N-Miss	8	4	12	
Yes	71 (50.0%)	44 (62.0%)	115 (54.0%)	
No, too young	68 (47.9%)	27 (38.0%)	95 (44.6%)	
No, decided not to tell him/her	3 (2.1%)	0 (0.0%)	3 (1.4%)	
**Caregiver Sex**				0.096^a^
Mother or Primary Female Caregiver	122 (81.3%)	69 (92.0%)	191 (84.9%)	
Father or Primary Male Caregiver	22 (14.7%)	4 (5.3%)	26 (11.6%)	
Other	6 (4.0%)	2 (2.7%)	8 (3.6%)	
**Caregiver Age**				1.000^b^
N-Miss	4	6	10	
Over 21	142 (97.3%)	68 (98.6%)	210 (97.7%)	
Under 21	4 (2.7%)	1 (1.4%)	5 (2.3%)	
**Caregiver Ethnicity**				0.582^b^
N-Miss	50	33	83	
Hispanic/Latino	2 (2.0%)	2 (4.8%)	4 (2.8%)	
Not Hispanic/Latino	98 (98.0%)	40 (95.2%)	138 (97.2%)	
**Caregiver Race**				0.862^b^
N-Miss	1	5	6	
Asian	1 (0.7%)	0 (0.0%)	1 (0.5%)	
Black/African American	140 (94.0%)	65 (92.9%)	205 (93.6%)	
Multi-Racial	3 (2.0%)	2 (2.9%)	5 (2.3%)	
White/Caucasian	4 (2.7%)	3 (4.3%)	7 (3.2%)	
Other	1 (0.7%)	0 (0.0%)	1 (0.5%)	
**Caregiver Marital Status**				< 0.001^b^, *
N-Miss	4	4	8	
Other	2 (1.4%)	0 (0.0%)	2 (0.9%)	
Married/Partnered	67 (45.9%)	12 (16.9%)	79 (36.4%)	
Single	67 (45.9%)	44 (62.0%)	111 (51.2%)	
Separated/Divorced	9 (6.2%)	13 (18.3%)	22 (10.1%)	
Widowed	1 (0.7%)	2 (2.8%)	3 (1.4%)	
**How far did caregiver get in schooling?**				0.029^c^, *
N-Miss	6	3	9	
Started School but Didn’t Finish	14 (9.7%)	12 (16.7%)	26 (12.0%)	
Finished High School/got GED	35 (24.3%)	21 (29.2%)	56 (25.9%)	
Started College or Trade School	33 (22.9%)	20 (27.8%)	53 (24.5%)	
Finished College or Trade School	49 (34.0%)	14 (19.4%)	63 (29.2%)	
Started Master’s or Doctoral Program	2 (1.4%)	2 (2.8%)	4 (1.9%)	
Finished Master’s or Doctoral Program	11 (7.6%)	3 (4.2%)	14 (6.5%)	
**Child Insurance**				0.152^a, f^
N-Miss	1	2	3	
Private Insurance	25 (16.8%)	7 (9.6%)	32 (14.4%)	
Military Health Care	1 (0.7%)	0 (0.0%)	1 (0.5%)	
Medicaid	120 (80.5%)	65 (89.0%)	185 (83.3%)	
None	3 (2.0%)	1 (1.4%)	4 (1.8%)	

^a^Chi-Square

^b^Fisher’s Exact Test

^c^Mantel-Haenszel Chi-Square

^d^One Way ANOVA

^e^Yes vs. No

^f^Private vs. Non-Private Insurance

^*^p < 0.05

### Demographics for unique caregivers

Mothers and female caregivers made up 86.7% of the those completing a PAT. Most caregivers were Black, single, and over the age of 21. In terms of education level, 84.4% of caregivers had finished high school, and 36% had finished college or obtained a master’s degree or more (Table 1). There was no significant difference between the Universal and Targeted/Clinical groups in caregiver sex, age, ethnicity, or race. Caregivers in the Universal group were more likely to be married or partnered than caregivers in the Targeted/Clinical group (*p* < 0.001). Caregivers in the Universal group were more likely to finish college or a masters/doctoral degree (*p* = 0.029).

### PAT scores and categorization

According to the score thresholds set by the PAT, 66.7% (*n* = 150) of patients were in the Universal category, 27.6% (*n* = 62) were Targeted, and 5.8% (*n* = 13) were Clinical. The average total score was 0.87 and the median was 0.73, both in the Universal range. There were statistically significant differences between the Universal and Targeted/Clinical groups in family structure and resources (*p* < 0.001), social support (*p* < 0.001), patient problems for ages 2 and over (*p* < 0.001), sibling problems for ages 2 and over (*p* < 0.001), sibling problems for both age groups (*p* = 0.015), caregiver problems (*p* < 0.001), caregiver stress (*p* < 0.001), and family beliefs (*p* < 0.001) subscales (Table 2).

**Table 2:** Total PAT scores and subscale scores by PAT risk category

	**Universal (*N* = 150)**	**Targeted/Clinical (*N* = 75)**	**Total (*N* = 225)**	***p* value**^**a,b**^
**Total**				< 0.001^a^, *
Mean (SD)	0.53 (0.25)	1.55 (0.45)	0.87 (0.59)	
Range	0.00—0.99	1.00—2.78	0.00—2.78	
**Family Structure/Resources Subscale**				< 0.001^a^, *
Mean (SD)	0.28 (0.16)	0.37 (0.17)	0.31 (0.17)	
Range	0.00—0.67	0.00—0.83	0.00—0.83	
**Social Support Subscale**				< 0.001^a^, *
Mean (SD)	0.02 (0.08)	0.34 (0.40)	0.12 (0.28)	
Range	0.00—0.40	0.00—1.00	0.00—1.00	
**Patient (under age 2) Problems Subscale**				0.549^b^
Mean (SD)	0.02 (0.06)	0.03 (0.11)	0.02 (0.08)	
Range	0.00—0.30	0.00—0.50	0.00—0.50	
**Patient (age 2 and over) Problems Subscale**				< 0.001^a^, *
Mean (SD)	0.08 (0.10)	0.22 (0.21)	0.12 (0.16)	
Range	0.00—0.48	0.00—1.00	0.00—1.00	
**Sibling problems: under 2 ONLY Subscale**				0.486^b^
Mean (SD)	0.00 (0.01)	0.00 (0.00)	0.00 (0.01)	
Range	0.00—0.10	0.00—0.00	0.00—0.10	
**Sibling problems: 2 and over ONLY Subscale**				< 0.001^b^, *
Mean (SD)	0.01 (0.03)	0.06 (0.12)	0.02 (0.08)	
Range	0.00—0.25	0.00—0.55	0.00—0.55	
**Sibling problems: under 2 + 2 & over Subscale**				0.015^b^, *
Mean (SD)	0.00 (0.00)	0.02 (0.10)	0.01 (0.06)	
Range	0.00—0.00	0.00—0.71	0.00—0.71	
**Caregiver Problems Subscale**				< 0.001^a^, *
Mean (SD)	0.05 (0.10)	0.25 (0.22)	0.12 (0.18)	
Range	0.00—0.50	0.00—0.90	0.00—0.90	
**Caregiver Stress Reactions Subscale**				< 0.001^b^, *
Mean (SD)	0.01 (0.04)	0.08 (0.16)	0.03 (0.11)	
Range	0.00—0.25	0.00—0.75	0.00—0.75	
**Family Beliefs Subscale**				< 0.001^b^, *
Mean (SD)	0.07 (0.10)	0.19 (0.19)	0.11 (0.15)	
Range	0.00—0.70	0.00—0.70	0.00—0.70	

^a^One Way ANOVA

^b^Wilcoxon Two Sample Test

^*^p < 0.05

### Family resources

The Family Structures and Resources subscale had the highest mean score of all the subscales (Table 2). Approximately eighty-three percent of caregivers used their own car to get to appointments. In addition to or instead of using their own car, 19% of caregivers used a different form of transportation such as Medicaid, public transportation, or rides from others.

**Fig. 1:**
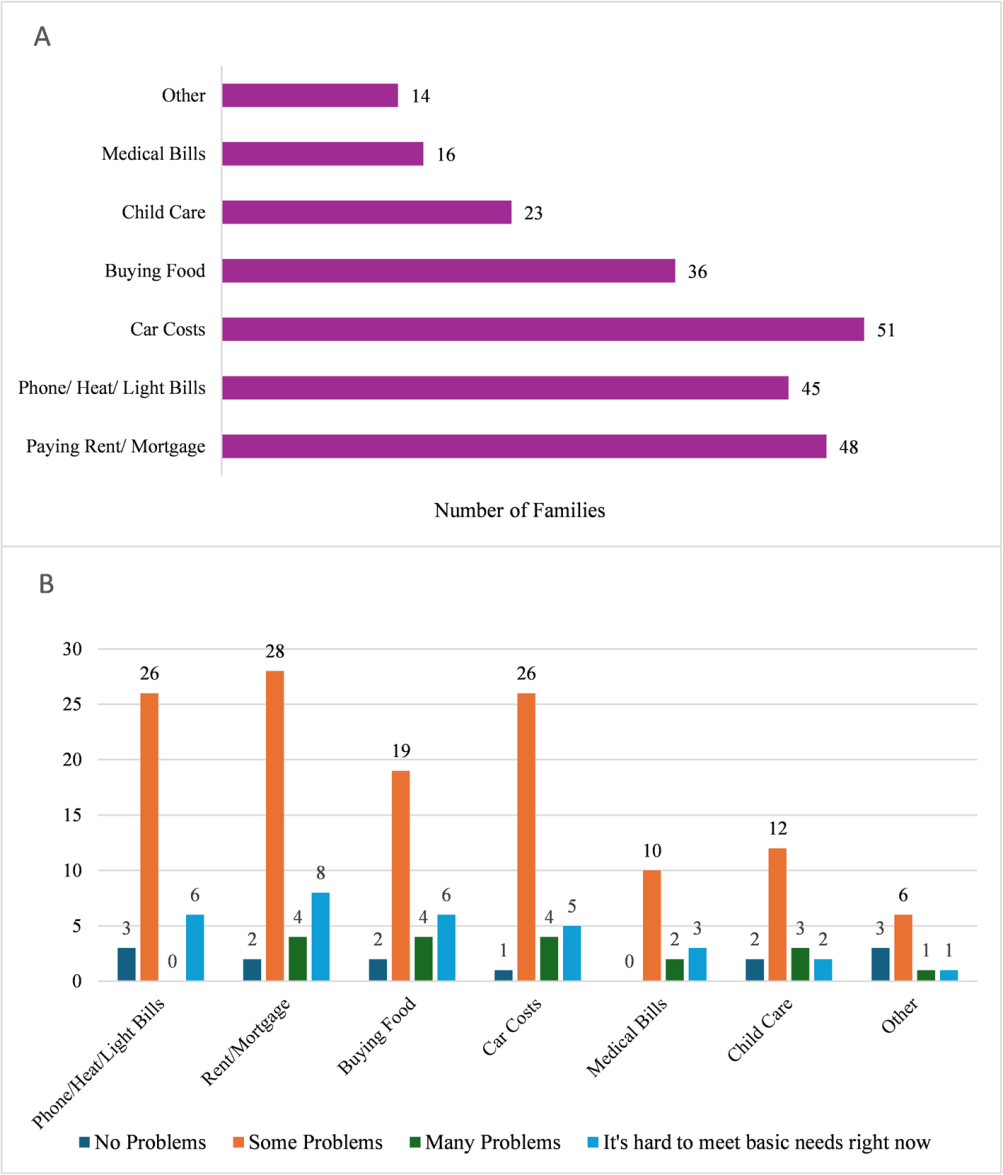
Financial difficulties endorsed by caregivers overall and by degree of financial concerns

Almost fifty percent of caregivers reported some form of financial difficulties, with 10.3% reporting many problems or being unable to meet basic needs (Table 3). The top three areas of money problems were car upkeep and gas (22.2%), paying rent/mortgage (20.9%), and phone/heat/light utilities (19.1%). Approximately seven percent of families who noted they had no financial difficulties proceeded to select categories of financial concern. About twenty-eight percent of families selected more than one category (Fig. 1).

**Table 3:** Degree of financial concerns by PAT risk category

	**Universal (*N* = 150)**	**Targeted/Clinical (*N* = 75)**	**Total (*N* = 225)**	***p* value**
**Is your family having money problems?**				< 0.001*a
N-Miss	6	4	10	
No problems	87 (60.4%)	21 (29.6%)	108 (50.2%)	
Some problems	47 (32.6%)	38 (53.5%)	85 (39.5%)	
Many problems	5 (3.5%)	7 (9.9%)	12 (5.6%)	
It’s hard to meet our basic needs right now	5 (3.5%)	5 (7.0%)	10 (4.7%)	

^a^Mantel-Haenszel Chi-Square

^*^p < 0.05

When stratified by PAT risk category, the majority of individuals in the Targeted/Clinical categories (greater total psychosocial risk) had some level of financial difficulty (*p* < 0.001) (Table 3). However, even in the Universal category (lesser total psychosocial risk), nearly 40% of families indicated some type of financial difficulty, including five that indicated they had trouble meeting their basic needs. About fifty-three percent of all patients with financial difficulties were still in the Universal category.

### Caregiver support

Support of the caregiver was determined for several categories (Table 4). For childcare and parenting, 51.6% of caregivers had support from a partner. For emotional support, 47.1% of caregivers had support from their partner, and 43.6% had support from other family members. For financial support, 17.8% of caregivers endorsed having no additional help. Almost 80% of all caregivers reported some level of help in all three categories.

**Table 4:** Sources of support endorsed by caregivers

	**n (%)**					
	**Childcare/ parenting (*N* = 206)**	**Emotional support (*N* = 194)**	**Money/ financial support (*N* = 189)**	**Help with information (*N* = 184)**	**Everyday tasks (e.g., meals, errands, rides) (*N* = 188)**	**Providing emotional or practical support to siblings of the patient (*N* = 182)**
**Spouse/partner**	116 (51.6)	106 (47.1)	97 (43.1)	82 (36.4)	98 (43.6)	97 (43.1)
**Child’s grandparents**	82 (36.4)	71 (31.6)	49 (21.8)	53 (23.6)	51 (22.7)	67 (29.8)
**Other family members**	87 (38.7)	98 (43.6)	59 (26.2)	68 (30.2)	70 (31.1)	84 (37.3)
**Friends**	52 (23.1)	77 (34.2)	33 (14.7)	56 (24.9)	46 (20.4)	57 (25.3)
**People at work**	3 (1.3)	10 (4.4)	6 (2.7)	17 (7.6)	4 (1.8)	5 (2.2)
**Church/spiritual community**	17 (7.6)	34 (15.1)	13 (5.8)	22 (9.8)	10 (4.4)	23 (10.2)
**Other**	11 (4.9)	4 (1.8)	6 (2.7)	23 (10.2)	10 (4.4)	8 (3.6)
**No one**	22 (9.8)	22 (9.8)	40 (17.8)	24 (10.7)	31 (13.8)	27 (12.0)

### Patient experience and vulnerability

Subsequent questions inquire about the experience, wellbeing, and vulnerability of the patient. The majority of caregivers reported their child did not seem sad, did not have developmental problems or other mental or medical health concerns, and had not been abused or the victim of a crime. The question of whether or not the child was moody garnered the most affirmative results, with 35.7% saying “yes” or “sometimes”. For patients under 2 years of age, the majority said they had no sleeping (81.8%) or feeding problems (80%). Approximately twenty-four percent of caregivers answered “yes” or “sometimes” about whether the child cries a lot. For children over 2, the category with the most positive results was whether the child got distracted easily, had trouble paying attention, or cries/gets upset easily with 40.3%, 32.3% and 32.3%, respectively, answering “yes” or “sometimes”.

### Sibling experience

**Fig. 2:**
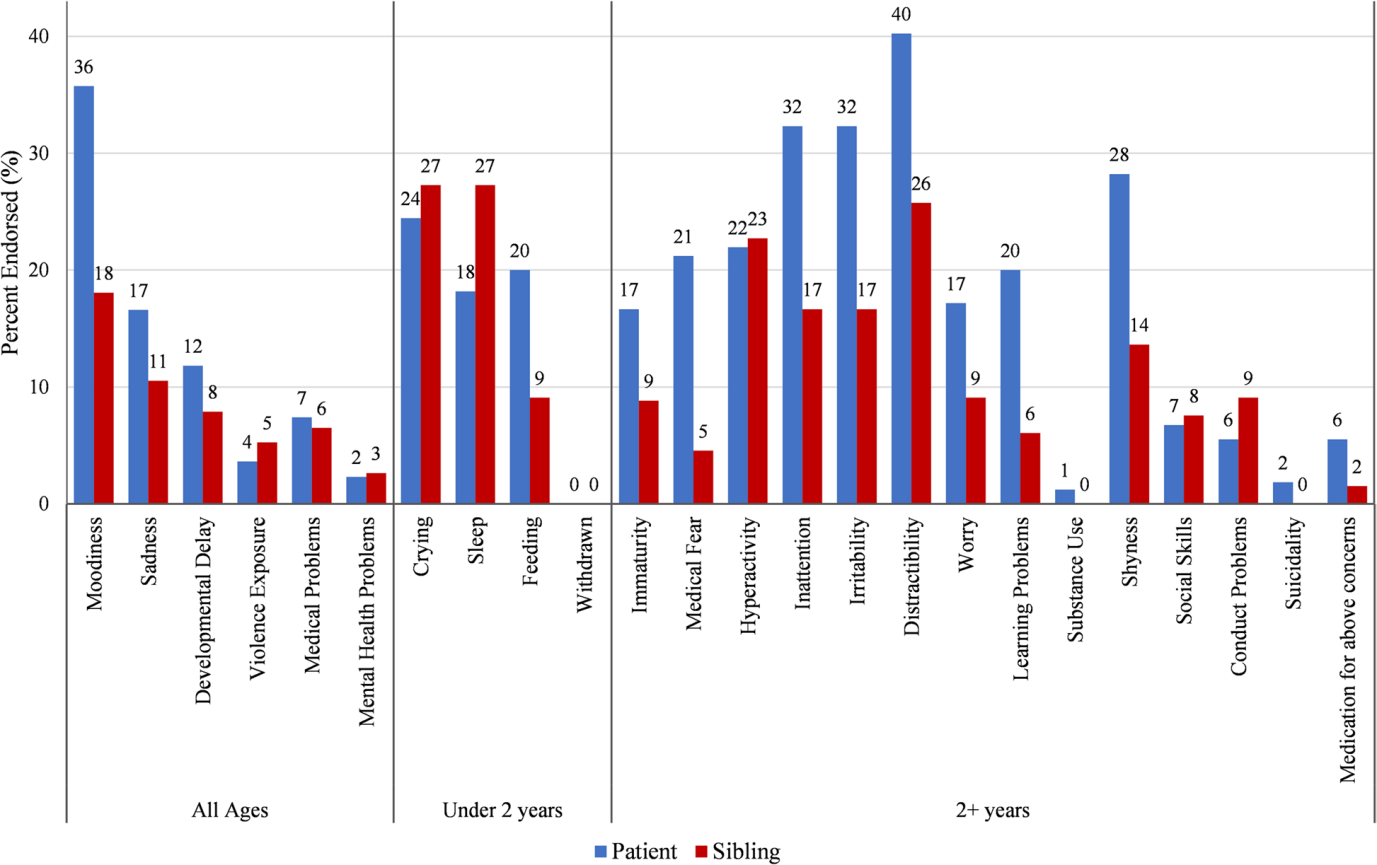
Patient versus sibling behaviors endorsed by caregivers

Approximately seventy-three percent of caregivers identified having other children living in the home, but only 77.6% of those caregivers answered questions related to the sibling experience. Of families who reported having other children in the home, approximately 95% identified having siblings over 2 years of age. Caregivers responded to the same questions about patient experience for the siblings. There was a higher number of patients affected than their siblings in 16 out of 24 categories. For example, only 17% of siblings had problems paying attention, compared to 32% of patients (Fig. 2).

### Caregiver experience and beliefs

Approximately twenty-three percent of caregivers reported worry or anxiety, and 24.7% reported feeling sad or depressed at times. Eleven percent of caregivers said they had problems paying attention or staying focused, and 14.7% reported a death in the family within the last year. Most caregivers did not have upsetting thoughts about their child’s illness (76.6%) or feel startled by reminders (86.0%), did not avoid reminders of the illness (94.9%), and did not lose interest in regular activities because of their child’s illness (87.9%). Only 3.8% of caregivers did not believe that the doctors and nurses would know how to help their child. Ninety-three percent of caregivers stated that it was mostly or very true that they were able to express their concerns with the medical team, and 95.2% believed it was mostly or very true that good treatment decisions would be made. Additionally, 85.7% of caregivers believed it was very true that they would be good parents through this experience and 85.8% did not believe that their situation was a “disaster.”

### Multiple PATs

Twenty-one caregivers completed multiple annual visits during the study period, and therefore, completed 2 PATs. No statistically significant differences were noted between the first and second PAT, except a slight decrease in the Family Structure Subscale Score (mean/SD; 0.28 (0.14) vs 0.22 (0.16), *p* = 0.048).

## Discussion

Children with SCD and their families experience significant health disparities, both related to disease sequelae and disparities in medical care, as well as social and environmental factors unrelated to their SCD. Large claims-based and national database studies have shown that less than 70% of children with SCD receive the recommended comprehensive care [3, 23]. A study done by our own group demonstrated significant caregiver-reported barriers to accessing care, most commonly resource-based barriers such as those assessed in the PAT [24]. Financial and resource scarcity, emotional and mental health burden, as well as neurocognitive effects of the disease are associated with poorer quality of life and health outcomes in SCD [25–27]. Provider bias and knowledge gaps diminish the quality of care patients with SCD receive, compounding the effects of resource and psychosocial barriers on disease management [5, 28]. Additionally, children with SCD and their caregivers are at increased risk for psychological distress, with parental stress impacting child health [11, 29, 30].

Universal screening for psychosocial risk using the PAT is a form of preventative health, allowing clinical teams to identify and provide directed social, environmental, and psychological resources for families to improve health outcomes for their child that may otherwise be incomplete with targeted assessments [31]. Two single institution studies validated the use of the PAT in cohorts of children and adolescents with SCD [16, 21], and our study provides the largest data regarding the universal clinical application of the PAT in a comprehensive SCD clinic.

While we found that most families in our study scored in the Universal category, nearly half of families in this group reported significant financial difficulties on the PAT, including several who did not endorse having any money problems on the survey, but subsequently selected several resource needs. This discrepancy was also noted in other studies with the PAT in SCD [16, 21]. Transportation, housing, and food insecurities are prevalent amongst families with children who have special healthcare needs, especially SCD, and may be overlooked if resources are distributed based on final PAT category alone. As such, a comprehensive review of the completed PAT assessment by a psychosocial team member, such as a Social Worker, Case Manager, or Community Health Worker is recommended to ensure such SDoH that may be impacting care access are identified and addressed.

Though universal screening for SDoH is becoming more prevalent in health systems, a similar breadth of assessment of caregiver or family stressors is not as common. Caregivers in our clinic endorsed significant psychosocial stress, including mental health concerns, on the PAT that may or may not be evaluated in other settings, particularly if caregivers do not have, or regularly see, their own healthcare providers [32]. While screening for post-partum depression is part of primary care guidelines for new mothers, continued, regular assessments of caregiver stress and psychological health are not standardized in pediatric care, particularly for caregivers of children with special healthcare needs [33], suggesting a significant gap in understanding for clinical teams of caregiver stress and its longitudinal impact on outcomes for children with SCD. Universal application of multidimensional assessments, such as the PAT, for families of children with SCD provides an opportunity for intervention by psychosocial team members to improve caregiver, and subsequently, patient health.

Caregiver responses to questions regarding their child and any siblings also provided insight into potential neurocognitive and behavioral concerns that may be impacting caregiver stress, as well as child health outcomes. For example, while caregivers did not endorse many psychological concerns for their child, they were most likely to endorse their child as seeming ‘moody’. This may be indicative of underlying mood concerns known to co-exist with SCD [34–36], but warrants assessing the patient’s perspective, as well, which is often more accurate for internalizing symptoms (e.g., anxiety, worries, hopelessness). Caregiver concern for externalizing behaviors, such as inattention or distractibility, were also reported at rates consistent with existing literature, as these are known difficulties for children with SCD [37, 38]. Notably, our sample also revealed higher levels of attention concerns for children with SCD compared to their siblings, suggesting that disease sequelae may pose a unique risk for this population.

Specific to our population, we noted a lower average total PAT score than other published studies in SCD [16, 21], with an increased degree of resiliency that drove this categorization, including positive views of the healthcare team, disease experience, and family beliefs. Specifically, average subscale scores for Family Structures, Social Support and Caregiver Stressors were lower. As a retrospective study, we cannot definitively say whether this increased resiliency is true broadly for families of children with SCD, specific to our population in our clinic, or even regionally defined. However, studies have shown that resilience factors can help navigate medical, psychosocial, and socioeconomic adversity for families of children with SCD [22]. Religious and spiritual coping, positive outlooks despite burdens, child autonomy, child participation in activities, and family connectedness all build resilience and help manage the stressors that can be associated with this diagnosis. Thus, not only do regular psychosocial assessments provide understanding of risk factors affecting individuals with SCD, but also potential protective factors [22, 39].

Limitations to our study include that it is a single institution study, though is representative of the patient population throughout our state as our catchment area encompasses the entire state, as well as includes a larger cohort than previously described PAT studies in SCD. Other limitations include those related to survey responses, as questions regarding ethnicity and number of individuals in the household were not always answered/answered correctly and required clarification by a team member. The lack of significance noted in the paired PAT analysis may be related to a small sample size or the limited time period; longitudinal analysis with the whole cohort will be important to understand any changes over time. Additionally, while the PAT may serve as a good screener for psychological and social/environmental concerns for children with SCD, it is limited to caregiver response which may differ from the child’s perspective of their experience.

## Conclusion

Families of children with SCD face significant barriers, both social and psychological, which can have a significant impact on health outcomes for their child. Universal screening for psychosocial risk factors in SCD with a comprehensive tool like the PAT, similar to other chronic disease populations, can potentially provide an opportunity to address concerns prior to its impact. Future studies should measure the impact of targeted interventions for psychosocial risk factors on healthcare utilization and caregiver and patient-reported outcomes, as well as consider the effectiveness of the PAT compared to multiple screening tools (e.g. SDoH screen, depression screen, etc.) in identifying social and psychological risk for patients with SCD and their families.

## Data Availability

All data generated or analyzed during this study are included in this published article.

## Abbreviations

SCD: Sickle Cell Disease
PAT: Psychosocial Assessment Tool
SDoH: Social Determinants of Health
PPPHM: Pediatric Psychosocial Preventative Health Model
EMR: Electronic Medical Record
